# Use of email in a family practice setting: opportunities and challenges in patient- and physician-initiated communication

**DOI:** 10.1186/1741-7015-4-18

**Published:** 2006-08-15

**Authors:** Ayaz Virji, Kimberly SH Yarnall, Katrina M Krause, Kathryn I Pollak, Margaret A Scannell, Margaret Gradison, Truls Østbye

**Affiliations:** 1 6-Step Weight Loss Center, 13191 Starkey Rd, Suite A-3, Largo, FL 33773, USA; 2 Department of Community and Family Medicine, Duke University Medical Center, Box 2914 DUMC, Durham, NC 27710, USA; 3 Johnston Memorial Hospital, PO Box 1376, 509 Brightleaf Boulevard, Smithfield, NC 27577, USA

## Abstract

**Background:**

Electronic mail (email) has the potential to improve communication between physicians and patients.

**Methods:**

We conducted two research studies in a family practice setting: 1) a brief, anonymous patient survey of a convenience sample to determine the number of clinic patients receptive to communicating with their physician via email, and 2) a randomized, controlled pilot study to assess the feasibility of providing health education via email to family practice patients.

**Results:**

Sixty-eight percent of patients used email, and the majority of those (80%) were interested in using email to communicate with the clinic. The majority also reported that their email address changed less frequently than their home address (65%, n = 173) or telephone number (68%, n = 181). Forty-two percent were willing to pay an out-of-pocket fee to have email access to their physicians. When evaluating email initiated by the clinic, 26% of otherwise eligible patients could not participate because they lacked email access; those people were more likely to be black and to be insured through Medicaid. Twenty-four subjects agreed to participate, but one-third failed to return the required consent form by mail. All participants who received the intervention emails said they would like to receive health education emails in the future.

**Conclusion:**

Our survey results show that patients are interested in email communication with the family practice clinic. Our feasibility study also illustrates important challenges in physician-initiated electronic communication. The 'digital divide' – decreased access to electronic technologies in lower income groups – is an ethical concern in the use of email for patient-physician communication.

## Background

With the rise of health care consumerism, patients have increasing expectations regarding the health care they receive. These expectations, coupled with the rising complexity of medical care and the increasing number of recommended preventive and chronic disease management services, have created a demand for more of physicians' time than is available [1,2]. Electronic communication technology could increase communication between the health care system and patients in general, enhance patient-physician interaction in particular, and provide opportunities to improve both the quality of care and the efficiency of clinical time use [3].

The use of electronic mail (email) among Americans has increased from 9% in 1995 to 74% in 2005 [4]. Consequently, email communication between patients and physicians is becoming an increasingly relevant possibility. In studies of primary care practices, more than half of surveyed patients had access to email or described themselves as email users, and a large proportion (70–90%) were interested in using email to communicate with their physicians [5-8]. Patients note speed, convenience, utility for managing simple problems, and avoidance of 'telephone tag' – the parties alternately leaving messages for each other – as clearly positive aspects of email [9]. However, patient-physician email use has not been widely adopted: only 5–10% of patients actually communicate with their doctors using this medium [6,10,11].

Physicians have been wary of adopting email as a major mode of communication with patients. They are concerned that lack of reimbursement, inundation with email, and dealing with trivial issues or topics inappropriate to email will only increase the time demands that they currently face [6,7,11]. Even among physicians who regularly use email, 25% are unhappy with using it, citing 'patient request' as the main reason for engaging in email contact [12]. These physicians, like those in other studies, report concerns about time demands, medicolegal risks, and the ability of patients to use email appropriately. Even the asynchronous nature of email, often cited as a benefit since it is available at the convenience of the user and does not require the arrangement of a scheduled appointment, can be seen as a potential detriment if used to replace appropriate face-to-face communication between patient and provider [13].

Reimbursement remains a problem; however, some studies indicate that the medium shows promise. Controlled trials have shown that physicians using email systems to communicate with patients spend only 5–10 minutes a day dealing with on average 12–13 emails a week [11,14,15]. Patients also tend to use the format appropriately by avoiding emergent issues, limiting the content to medical and business-related topics (e.g. appointment setting), and including only one request per email [16-18]. Also, while an early study showed no effect of an email system on physicians' efficiency [15], later studies using web-messaging demonstrated an increase in the number of patient visits and services provided per workday per physician, as well as a reduction in telephone calls from patients, resulting in a 10% increase in physicians' productivity [19,20]. Efficiency might be further enhanced by the use of non-physician staff to triage emails [21].

As physicians begin to adopt email communication with patients, it is of interest to learn the extent to which email can be used to improve medical care and to expand the possibilities email presents. For instance, while a number of studies have focused on patient-initiated communication, only one published report that we could identify has addressed the possibility of disseminating health-related information to established patients using email [22]. The use of electronic media to provide health education to patients could reduce the time needed during face-to-face patient visits for counseling and preventive education, which can be time-consuming [1,23]. To assess the possibilities for both patient-initiated and physician-initiated email communication, we conducted two research studies in a family practice setting to determine 1) the proportion of patients who would be receptive to communicating via email with their physician, and 2) the feasibility of providing preventive health education via email to patients.

## Methods

### Site

The Duke Family Medicine Center is an academic family practice located on the Duke University campus at Durham, NC, USA. In addition to Duke University employees, the practice accepts patients with most insurance plans, including Medicare and Medicaid, and averages 35,000 visits per year.

Email penetration among the family practice patients was determined by an anonymous survey of patients, conducted from November 2002 to March 2003. The feasibility of an email intervention designed to increase preventive screening and counseling services in women aged 18–25 and 50–65 years was evaluated from November 2001 to May 2002.

### Study 1: patient survey

A simple, anonymous patient survey evaluating the feasibility of electronic communication was distributed to a convenience sample of patients in the lobby of the Duke Family Medicine Center. On two designated days per week for a period of 12 weeks, the clinic staff gave patients with established appointments a survey while they were signing in, to complete and return to a box in the lobby. To avoid duplicate responses from the same patient on different days, the first question asked if the respondent had completed the survey previously; if so, they were asked to mark 'yes' to the question and deposit the survey in the collection box.

A screening question asked if the patient had an email address; if they did, they were instructed to complete the remaining questions. These questions included whether patients were actively using email (defined as checking it at least weekly), stability of email address compared with home address and telephone number, interest in clinical email, and willingness to pay for email access to physicians. The survey was designed to be brief and not interfere with patient flow, and required about 3 minutes to complete. The study was approved by the Duke University Institutional Review Board.

### Study 2: health education and preventive services feasibility study

The purpose of the second study was to determine the feasibility of providing preventive counseling and screening (where appropriate) to patients in advance of their scheduled preventive maintenance exams, with the goal of increasing preventive services delivered while decreasing time spent by physicians. With prior education and screening, patients could theoretically be prepared with questions, and test results could be available at the time of the visit. The primary goals of the study were to determine the feasibility of implementing a patient education intervention via email, and determine whether patients would be receptive to receiving such information via email. Secondary goals were to determine whether the intervention increased the screening and counseling services delivered in the preventive care encounter.

Preventive screening and counseling services were chosen for this study from the US Preventive Services Task Force Recommendations. Because of the limitations of email, only certain counseling and screening procedures were appropriate; these generally were services for women in certain age groups and included counseling on contraception, protection against sexually transmitted diseases, smoking cessation, calcium intake, and hormone replacement therapy. Screening tests available included a urine test for chlamydia, fecal occult blood testing, and mammography.

Women aged 18–25 and 50–65 were identified via the scheduling computer system five weeks prior to a preventive care office visit and were mailed an introductory letter and consent form. These potential participants were then contacted by a trained telephone interviewer, and eligible women (who had email access and were willing to participate) completed a baseline survey that assessed risk behaviors relating to condom use, smoking, and calcium intake, and last receipt of relevant screening tests, as well as participants' demographics such as race and insurance status. Participants were randomized to intervention or control groups and their medical charts were reviewed (to confirm screening test status) after the written consent form was returned by mail.

The intervention group received two emails that addressed screening tests and counseling relevant for that patient. The emails were tailored to individual risk assessments based on the baseline survey and chart review. Screening was scheduled and performed at the clinic, and the counseling text was compiled by the study physician from existing patient education materials. The control group received two emails with general practice information. After the office visit, all study participants received an email follow-up survey assessing the screening and counseling services delivered and patients' satisfaction with the email intervention, as well as $25 compensation.

Microsoft Access and SAS version 8.0 (Cary, NC, USA) were used for data management and analysis. The study was approved by the Duke University Institutional Review Board.

## Results

### Study 1: patient survey

Results of the email penetration study are presented in Table 1. Approximately 700 surveys were distributed, of which 474 were returned to the collection box. Of these, 84 were excluded as duplicate responses, leaving an analysis sample of 390 surveys. At the Duke Family Medicine clinic, 68% of patients surveyed (n = 266) actively used email. Of these 266 patients, the majority reported that their email address changed less frequently than their home address (65%, n = 173) or telephone number (68%, n = 181). In addition, there was a strong interest among patients (80%, n = 212) in communicating with the clinic via email. Forty-two percent of patients with email (n = 111) were willing to pay 'a small annual fee' to have email access to their physicians.

### Study 2: health education and preventive services feasibility study

Characteristics of the subjects in the preventive education feasibility study are presented in Table 2. Sixty-eight women in the defined age groups with scheduled appointments were identified during the study period. Of the 53 who were reached by telephone for the baseline survey, 26% (n = 14) did not have email. Relative to their representation in the overall sample, black women were notably less likely than white women to be eligible because they lacked access to email. Older women were slightly less likely to have email than younger women, and those with Medicaid were less likely to have email than those with private insurance. Twenty-four women agreed to participate and completed the baseline survey. However, of those, 25% (n = 6) did not return the consent form in time to participate in the study, despite an enclosed stamped self addressed envelope and reminder phone call. Eighteen women received the study emails, 17 kept their preventive health appointment, and of those, 16 filled out the final email survey (18–25 group: intervention n = 2, control n = 2; 50–65 group: intervention n = 5, control n = 7).

Because of the difficulties in recruiting and maintaining participants, there is limited ability to compare secondary outcomes in the intervention and control groups. Regardless, according to follow-up survey responses there appeared to be little difference by arm in the number of topics covered in the preventive health visit or in the receipt of screening tests, although intervention group women aged 50–65 were more likely than controls (4 of 5 vs 4 of 7) to have received fetal occult blood test screening. More relevant to the primary aims, all respondents in the intervention arm (both 18–25 and 50–65 age groups) said they would like to continue receiving health education emails in the future.

## Discussion

Our survey results look promising for the future utilization of patient-initiated electronic communication in family practice clinics. Sixty-eight percent of respondents had email access, and 80% were interested in using email to communicate with their doctors. Forty-two percent of the patients with email would be willing to pay a small annual fee to have electronic access to their physician, similar to what has been reported in the general population (37%) [8]. In the future, the introduction of a clinical procedure code for email communication could make it possible for time spent using email to be reimbursed, which, coupled with appropriate billing systems, could spur increased utilization.

Respondents also reported that their email addresses were fairly stable, and in many cases less likely to change than their other contact information. This finding suggests that it may be in the interest of clinics to not only develop email capabilities but to collect email addresses from patients as part of their standard personal contact information.

Our prevention education feasibility study, however, demonstrates some of the issues involved in physician-initiated electronic communication. From a research perspective, beyond the usual difficulties in establishing initial contact and study refusals, studies based on electronic communication lose potential participants because of lack of access to email and difficulties in the paper-based consent process. One-quarter of recruited participants failed to return the consent form, indicating that a mail-based element in an otherwise electronic intervention can have a strong negative impact on recruitment. Salient to both research and to regular clinical communication, of the few women enrolled in the trial, two had problems receiving the emails sent from the clinic: multiple messages bounced back with the explanation that the 'mailbox was full'. When dealing with email from the physician to the patient, the patients may be less concerned with technological issues than if they initiate the contact themselves.

Roughly 76% of women recruited for the prevention study had access to email, a slightly higher number than we found in the general population. However, this is not unexpected, as the population attending preventive visits would be likely to have private insurance and higher levels of income and education, which are positively associated with Internet access [24]. Conversely, it is important to note that women of lower socioeconomic status, as indicated by enrollment in Medicaid, were disproportionately ineligible to take part in the study because of their lack of access to email. This issue of the 'digital divide' – decreased access to electronic technologies in lower income groups – is an ethical concern in the use of email for patient-physician communication [13,25]. Our study showed that Medicaid patients and black patients were less likely to have access to email, and that these groups also had a higher mean number of annual visits to the clinic. They probably represent a less healthy and less affluent subgroup of our patient population, and yet they would have had access neither to patient-initiated communication nor to the health education resources initiated by the clinic.

Our patient survey study was limited by certain aspects of the design and the use of simple questions. Regarding study design, it would have been helpful to collect demographic information on those patients identified as not having an email address. This would have provided additional insight into the influence of age, income, and gender on access to email in our patient population. Also, we chose to utilize dichotomous questions for our survey instead of Likert scales. Although Likert scales, which allow the respondent to indicate levels of agreement or disagreement, would have provided more detail about 'interest level' in our patients, we used dichotomous questions to minimize the time required to complete the survey in our waiting room, and also to avoid the central tendency bias and acquiescence bias common to Likert questions [26].

Our study on the feasibility of clinic-initiated email was limited by the small number of patients completing the study and by a single clinic site. The study did not yield the results we hoped for, as recruitment was hampered by numerous issues unrelated to email access. Despite these limitations, both studies highlight the growing interest in a new method of patient-physician communication, and present potential issues for those wishing to use the internet for research and for patient education.

The ethical and legal ramifications of electronic communication with patients should be thoroughly understood prior to widespread use by individual medical physicians or practices. The American Medical Informatics Association recommends the standard use of informed consent, which should include itemized terms of communication guidelines, instructions for when to 'escalate' to phone calls and office visits, description of security mechanisms in place, and indemnity of the health care institution for information loss due to technical failure [27].

Case law regarding the use of clinical emails with patients is not yet well developed. Physicians should proceed with caution, particularly when giving medical advice or responding to unsolicited emails from patients that may initiate an 'implicit contract' [28]. In addition, many areas of the country require out-of-state physicians to be licensed in the state where the care will be administered before they may give recommendations via email [29]. The Health Insurance Portability and Accountability Act's requirements of encryption software to protect patient confidentiality should be in place prior to initiating any type of medical-based electronic communication.

## Conclusion

Email communication has the potential to improve patient access to healthcare, reduce administrative costs, and improve patient satisfaction. Access to email is likely to increase with new technology and lower costs and may be a better way to reach patients, particularly if email addresses change less often than street addresses and phone numbers. Among those women who participated in our feasibility study and received emailed health education materials, there was a high level of interest in receiving future emails from the clinic. This approach to using email – to initiate health-related information exchange from the clinic to the patient – has not been widely reported. We believe this approach is promising but will need to be further developed and refined.

## Competing interests

The author(s) declare that they have no competing interests.

## Authors' contributions

AV (study 1) and KSHY (study 2) conceived of and developed the studies described and drafted the manuscript. KMK assisted with drafting the manuscript and coordination of the two studies. KIP assisted in the design of study 2 and writing the manuscript. MAS assisted in the design of study 1 and writing of the manuscript. MG assisted in the design of study 1 and writing of the manuscript. TØ assisted in the design of both studies and writing of the manuscript. All authors read and approved the final manuscript.

## Pre-publication history

The pre-publication history for this paper can be accessed here:



## Figures and Tables

**Table 1:** Results of email usage and preferences survey of 390 patients at the Duke Family Medicine Center

Do you have an e-mail address? (if no, please stop now and hand in form at front desk)	Yes = 266 (68)
*Total respondents for remaining questions:*	*266 (100%)*
Do you check your e-mail at least weekly?	Yes = 247 (93)
Does your e-mail address change (more/less) often than your home address?	Less often = 173 (65)
Does your e-mail address change (more/less) often than your phone number?	Less often = 181 (68)
Would you be interested in using e-mail to communicate with Duke Family Medicine personnel?	Yes = 212 (80)
Would you be willing to pay a small annual fee to have e-mail access to your doctor for health care advice?	Yes = 111 (42)
Would you say the total yearly income of your household is:	
less than or equal to $15,000	12 (5)
$15,001–$30,000	61 (23)
$30,001–$45,000	53 (20)
>$45,000	109 (41)
don't know	13 (5)
refused	18 (6)
What is your age group?	
18–45	147 (55)
46–65	7 (3)
65+	97 (36)
refused	15 (6)

Values are number (%).

**Table 2:** Characteristics of women in the preventive education feasibility study

**Characteristic**	**With appointments**	**Completing baseline survey**	**Not eligible**	**Not reached**	**Refused**
	(n = 68)	(n = 24)	(n = 14)	(n = 16)	(n = 14)

Age					
18–25	17 (25)	8 (33)	3 (21)	5 (31)	1 (7)
50–65	51 (75)	16 (67)	11 (79)	11 (69)	13 (93)
Race					
Asian	1 (2)	0 (0)	0 (0)	1 (6)	0 (0)
Black	26 (38)	7 (29)	8 (57)	3 (19)	8 (57)
Hispanic	1 (2)	0 (0)	1 (7)	0 (0)	0 (0)
White	37 (54)	15 (63)	5 (35)	11 (69)	6 (43)
Unknown	3 (4)	2 (8)	0 (0)	1 (6)	0 (0)
Insurance					
Private	62 (91)	21 (88)	12 (86)	16 (100)	13 (93)
Medicare	3 (4)	1 (4)	1 (7)	0 (0)	1 (7)
Medicaid	1 (2)	0 (0)	1 (7)	0 (0)	0 (0)
Unknown	2 (3)	2 (8)	0 (0)	0 (0)	0 (0)
Appointments/year, mean (SD)	5.3 (4.6)	4.4 (4.2)	8.3 (6.4)	4.3 (3.8)	5.0 (3.0)

Values are number (%) except where indicated otherwise.
